# Integrated computer analysis and a self-built Chinese cohort study identified GSTM2 as one survival-relevant gene in human colon cancer potentially regulating immune microenvironment

**DOI:** 10.3389/fonc.2022.881906

**Published:** 2022-10-03

**Authors:** Wei Zhang, Yutong Shi, Shumeng Niu, Lintai Li, Liewen Lin, Xucan Gao, Wanxia Cai, Yumei Chen, Yafang Zhong, Donge Tang, Min Tang, Yong Dai

**Affiliations:** ^1^ Clinical Medical Research Center, Shenzhen People’s Hospital, The Second Clinical Medical College of Jinan University, Shenzhen, China; ^2^ South China Hospital, Health Science Center, Shenzhen University, Shenzhen, China; ^3^ Medical School, Nanchang Institute of Technology, Nanchang, China; ^4^ Key Laboratory of Diagnostic Medicine designated by the Chinese Ministry of Education, Chongqing Medical University, Chongqing, China; ^5^ Laboratory Department, Shanghai Hongkou Jiangwan Hospital, Shanghai, China

**Keywords:** GSTM2, immune microenvironment, prognostic biomarker, colon cancer, RAD21

## Abstract

According to a recent report by GLOBOCAN, colorectal cancer is the third most common and second most deadly cancer in 2020. In our previous proteomic study, we found that the expression of GSTM2 in colon tissues was significantly lower than that in para-cancer tissues, and its lower expression was associated with reduced overall survival rate of patients, suggesting that this gene might play a role in the occurrence of colon cancer. As a member of the detoxifying enzyme family, GSTM2 is likely to play an important role in the initiation of tumors. Whereas, the functions of GSTM2 in colon cancer are barely known. In this study, using the RNA-Seq datasets of colon cancer patients from public database (n_tumor_ = 457, n_normal_ = 41), we confirmed the reduced expression of GSTM2 and its prognostic value in colon cancer. Furthermore, we used our own Chinese cohort (n_tumor_ = 100, n_normal_ = 72) verified the lower GSTM2 expression in colon cancer, and also its effects on patient prognosis. Subsequently, we uncovered two potential reasons for the lower expression of GSTM2 in colon cancer tissues, including the deep deletion of GSTM2 on genome, and the up-regulation of RAD21 or SP1. Moreover, we disclosed that GSTM2 might be involved in several immune-related pathways in colon cancer, such as chemokine signaling and leukocyte transendothelial migration. Finally, we revealed that the GSTM2 expression was closely related to the immune-related scores of colon cancer and the infiltration ratios of various immune cells, suggesting that GSTM2 might regulate the development of colon cancer by modulating immune microenvironment. In conclusion, we uncovered the prognostic value of GSTM2 based on the public data and our own data, revealed its potential regulatory role in tumor immune microenvironment, and disclosed the probable reasons for its lower expression in colon cancer. The findings of our study provide a potential prognostic biomarker and drug target for clinical diagnosis and treatment of colon cancer.

## Introduction

According to the latest cancer statistics, the incidence and mortality rate of colorectal cancer is the third and second highest in the world, respectively. Globally, about 1.93 million people have been diagnosed as colorectal cancer, with even more than 930,000 deaths (1). In addition to surgical treatments, radiation, and chemotherapy, the treatments with immune checkpoint inhibitors (ICIs) have achieved success in the therapy of colorectal cancer in recent ten years (2). Many studies have revealed that the use of PD-1 and CTLA-4 inhibitors is effective in advanced colon cancer patients with mismatch repair defects or highly unstable microsatellites. However, most colon cancer patients are in a non-mismatched repair state, or a microsatellite stable state, thereby may not benefit from the ICI treatment (3). Therefore, it is necessary to continue exploring novel immune drug targets for colon cancer treatment.

Recently, numerous studies have revealed that tumorigenesis is strongly associated with the composition of immune microenvironment (TIM), such as immune cells and relevant cytokines (4). There is growing evidence that the TIM is valuable in predicting patient prognosis in multiple cancers (5). Different types of immune cell infiltration may provide a favorable or unfavorable environment for cancer development, and also being one of the key factors for immunotherapy responses.

The glutathione-S-transferase family (GST) is an important antioxidant enzyme family in living organisms, and GSTM belongs to the Mu subfamily of the GSTs (6). The GSTMs encode a series of detoxification enzymes, participating in detoxifying electrophilic compounds through coupling glutathione (carcinogens, environmental toxins, drugs, and oxidative stress products, etc.) (7). Some studies have demonstrated that GSTM1, GSTA4, GSTO1, in addition to their protection against oxidative stress, participate in the processes including immune defense and antiviral response (8–10). However, there are few studies reporting on the GSTM’s functions towards the TIM, and so as their actions on the tumor prognosis.

In our previous proteomic study (n_tumor_ = 8, n_normal_ = 8), we observed an extreme decrease in the expression of GSTM2 in colon tumor tissues versus normal adjacent tissues (11). Meanwhile, the data set from Clinical Proteomic Tumor Analysis Consortium (CPTAC) further confirmed our finding, and revealed that the reduced GSTM2 protein expression was correlated with lower patient survival rate (Wei Zhang, et al). Therefore, we hypothesized that GSTM2 might play a role in the tumorigenesis of colon cancer. A previous study has proclaimed that the butyrate can induce the GSTM2 expression in colon cancer, implying that GSTM2 may increase the detoxification ability of colon mucosa and play a protective role (12). Meanwhile, the mRNA expression of GSTM2 has also been found to be associated with prognosis of colon cancer patients based on a bioinformatics analysis (13), but its protein expression has not been verified in a self-built cohort, either its potential functions and mechanisms underlying tumorigenesis not explored in depth.

In this study, we studied the mRNA and protein expression of GSTM2 in colon cancer tissues and normal colon tissues using the public data sets. Besides, we analyzed the effects of GSTM2 on patient survival based on three independent data sets. Further, we revealed the possible pathways by which GSTM2 affected the colon cancer tumorigenesis, and investigated the correlation between the GSTM2 expression and the infiltration ratios of immune cells. Moreover, we explored the probable reasons for the decreasing expression of GSTM2 in colon cancer. Finally, we validated the GSTM2 expression and its action on patient survival based on a self-built Chinese cohort (n_tumor_ = 100, n_normal_ = 72).

## Materials and methods

### Expression spectrum

Tumor Immune Estimation Resource (TIMER) is a comprehensive database containing RNA-Sequencing data sets from The Cancer Genome Atlas (TCGA) and The Genotype-Tissue Expression (GTEx), used for the analysis of the gene expression and immune cell infiltration (14). In our study, the “Diff Exp” module was used to investigate the GSTM2 expression in multiple types of cancers and normal tissues.

### The relationship between GSTM2 expression and clinical features

UALCAN is a comprehensive interactive database providing in-depth analysis of data sets from TCGA and Clinical Proteomic Tumor Analysis Consortium (CPTAC) (16). In this study, the mRNA and protein expression of GSTM2 was analyzed using the UALCAN database. Besides, the relationship between the GSTM2 expression and the clinicopathological parameters (sex, age, ethnicity, tumor stage, body weight, histological typing, lymph node metastasis, and TP53 mutation) in colon cancer patients were also explored using the UALCAN database.

### Survival analysis

Prognoscan is an integrated database including the publicly available chip data that is used to analyze the actions of gene expression on patient prognosis (17). In this study, the effects of GSTM2 expression on patient survival was investigated using the microarray data sets of colorectal cancer in the Prognoscan database.

### Immunological analysis

XCELL, TIMER, CIBERSORT, MCPCOUTER, QUANTISEQ, and EPIC are the algorithms that estimate the proportion of immune cells in tumors based on the gene expression matrices (18). This study used these six algorithms to analyze the correlation between the GSTM2 expression and the ratios of different infiltrating immune cells. Using the “Immune” module on the TIMER 2.0 website, the link between GSTM2 expression and immune infiltration ratios was investigated. SangerBox was used to study the relationship between GSTM2 expression and microsatellite instability (MSI).

### Genetic mutations

The cBioportal database is an open platform for exploring gene mutations in multiple cancers (19). In this study, five independent studies were selected to search the GSTM2 mutations in colon cancer patients. The “cancer types summary” module was utilized to explore the frequency of GSTM2 mutations in colorectal cancer. Through the “mRNA vs. Dx” in “Plots” module, the relationship between the GSTM2 expression and different types of GSTM2 mutations in colon cancer was analyzed.

### Transcription factors

The hTFtarget database is a large-scale database collecting most human transcription factors and their experimentally confirmed target genes in multiple organs (20). In our study, the transcription factors of GSTM2 in colon tissue was searched using the hTFtarget database.

### Correlation analysis

Gene Expression Profiling Interactive Analysis (GEPIA) provides online interaction and customization analyses, including expression profiling of tumor and normal tissues (15). The “Correlation Analysis” function in the GEPIA database was used to investigate the expression correlation between two genes. A p-value < 0.05 was regarded as statistically significant. In this study, the data sets of colon adenocarcinoma (COAD) and normal samples from TCGA and the data sets of sigmoid colon normal tissue samples from GTEx were used.

### Gene set enrichment analysis (GSEA)

The co-expressed mRNAs of GSTM2 in colorectal cancer were obtained from the Linkedomics database, and the “LinkInterpreter” function in this database was used to investigate the potential relevant pathways of GSTM2 in colon cancer based on GSEA.

### Single sample gene set enrichment analysis (ssGSEA)

For colon cancer, RNAseq data (level 3) and clinical data were retrieved from TCGA database. The R program GSVA package was used for analysis, and the method “ssGSEA” was chosen. Finally, spearman correlation analysis was applied to examine the correlation between GSTM2 expression and the pathway scores. Statistics were judged significant at p value 0.05.

### Patients

The paraffin blocks of colon cancer tissues were provided by the National Human Genetic Resources Sharing Service Platform. The inclusion criteria were as follows: age ≤ 90 years old, histologically-confirmed colon cancer, no serious major organ dysfunction, and no chemotherapy or radiotherapy. The exclusion criteria were: age ≥ 90 years, severe major organ dysfunction, prior chemo- or radiation treatment, or a family history of colon cancer. All patients were informed and had signed an informed consent form. This work was carried out under the supervision of the Ethics Committee of Taizhou Hospital and met the requirements of the World Medical Association for the execution of human experiments.

### Immunohistochemistry assay

The tissue sections were stayed at room temperature for 60 minutes and then soaked in xylene for 10 minutes. Then, the soaking step was repeated once. Next, the chips were dewaxed in 100%, 95%, and 75% ethanol, respectively, and heated at 95°C for 15 minutes in the 0.01 M lycium buffer (pH = 6.0). This heating step was repeated 1-2 times. The chip was then cleaned three times with the PBS solution for 5 minutes each. Subsequently, 3% H2O2 was dripped onto the chip, and the chip was stayed at room temperature for 10 minutes, and then washed three times with the PBS solution for 5 minutes each. Subsequently, the chip was incubated with the blocking solution for 20 minutes at room temperature. The primary antibody of GSTM2 (Invitrogen, MA5-29311, 1:1500) was then added to the chip and the chip was incubated at 4°C for 12 hours. Then, the chip was cleaned three times with the PBS solution for 5 minutes each. The incubation solution with secondary antibody was then added to the chip, and the chip was incubated at 37°C for one hour. Then, the chip was cleaned three times with the PBS solution for 5 minutes each. Next, the diaminobenzidine (DABs) was used to color the chips, and the staining intensity was observed under a microscope. Subsequently, the chip was rinsed with the tap water for 10 minutes, and incubated with hematoxylin for 2 minutes, and differentiated with hydrochloride alcohol. After washed with the tap water for 10 minutes, the chip was then dehydrated, transparent, and sealed. Next, the stained chip was scanned using the Aperio Scanner (LEICA, Aperio XT), and the staining intensity and the proportion of the stained cells were assessed by two pathologists. 0, 1, 2, and 3 were used to represent the staining strength (0 represented negative, 1 represented weak, 2 represented moderate, and 3 represented strong). The proportion of the stained cells was evaluated based on the percentage of the positively stained area relative to the entire cancer region. Finally, the numbers representing the staining intensity and the proportion were multiplied to obtain the staining score.

### Statistical analysis

GraphPad Prism 6.0 was used for the statistical analysis. The GSTM2 expression in cancerous and normal tissues were assessed using the unpaired T-test. The survival analysis was performed using the “Survival” model of GraphPad Prism 6.0. The “Kaplan Meier Survival Curve” module in Sangerbox was used to determine the best cutoff value of GSTM2 expression in clinical samples. The Cox regression analysis was performed using SPSS Statistics 27. A p value < 0.05 was considered statistically significant, and the confidence interval was set as 95% confidence.

## Results

### GSTM2 is decreasingly expressed in colon cancer tissues versus normal tissues

**Figure 1:**
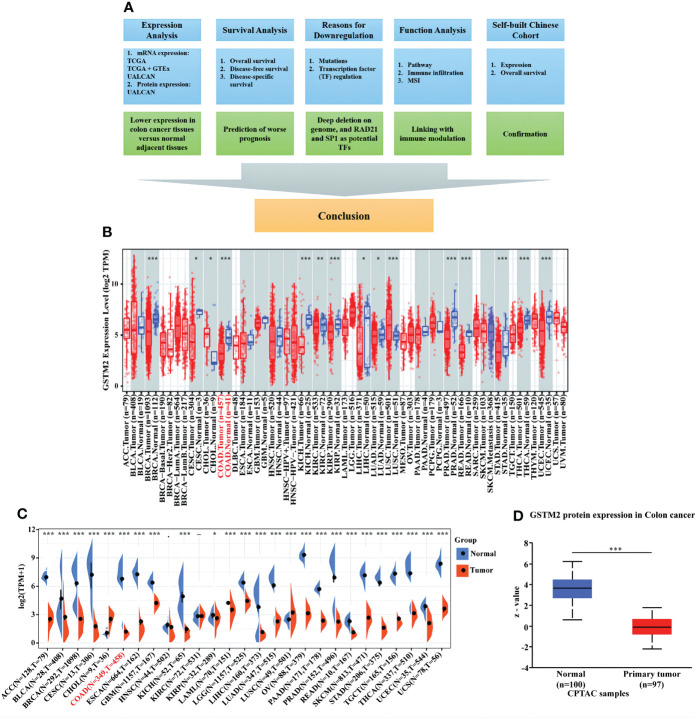
GSTM2 is Decreasingly Expressed in Colon Cancer Tissues versus Normal Tissues. **(A)** Flow chart of this research. GSTM2 mRNA expression in different types of cancer was studied using **(B)** the TIMER database, and **(C)** the TCGA database and GTEx database. The protein expression of GSTM2 in colon cancer tissues versus normal tissues was investigated using **(D)** the UALCAN database. *p < 0.05, **p < 0.01, and ***p < 0.001.

To explore the potential role of GSTM2 in tumorigenesis, we first used the TIMER database to assess the mRNA expression of GSTM2 in various human tumors. The results showed that GSTM2 was differentially expressed in tumor tissues versus normal tissues in 15 tumors, showing a down-regulated expression in 13 cancers, including COAD, breast invasive carcinoma (BRCA), cervical squamous cell carcinoma and endocervical adenocarcinoma (CESC), kidney chromophobe (KICH), kidney renal clear cell carcinoma (KIRC), kidney renal papillary cell carcinoma (KIRP), liver hepatocellular carcinoma (LIHC), lung adenocarcinoma (LUAD), prostate adenocarcinoma (PRAD), rectum adenocarcinoma (READ), stomach adenocarcinoma (STAD), thyroid carcinoma (THCA), and uterine corpus endometrial carcinoma (UCEC) (**Figure 1B**). In addition, after analyzing the data sets from TCGA and GTEx, we found that the GSTM2 expression was down-regulated in 23 tumors and only up-regulated in two tumors, and most of the results were consistent with the results of **Figure 1B** (**Figure 1C**). Furthermore, we investigated the GSTM2 protein level using the data set from CPTAC and discovered that the GSTM2 protein expression in colon cancer tissues was also reduced compared to normal colon tissues (n _tumor_ = 97, n _normal_ = 100) (**Figure 1D**).

### The Correlation between the GSTM2 expression and various clinical features

**Figure 2:**
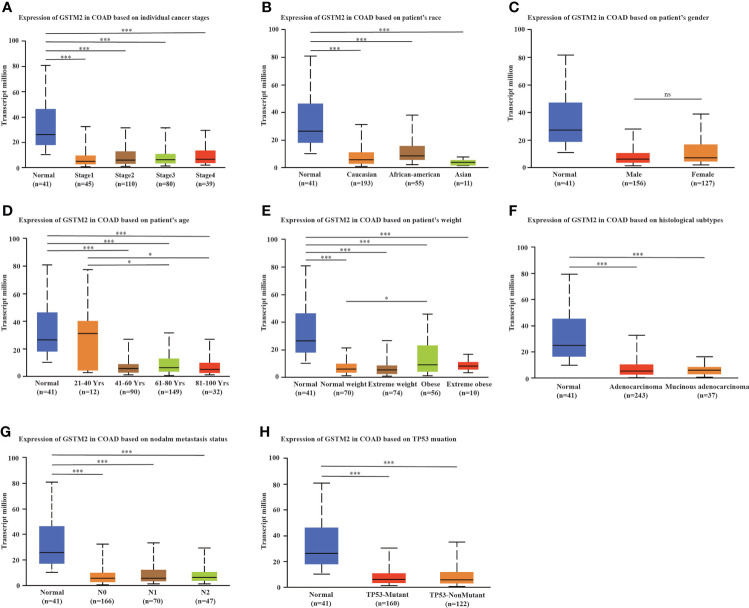
The Correlation between the GSTM2 Expression and Various Clinical Features. Analysis revealed the correlation between GSTM2 expression and **(A)** tumor stages, **(B)** race, **(C)** gender, **(D)** age, **(E)** weight, **(F)** histological subtypes, **(G)** metastasis, and **(H)** TP53 mutation. N0 indicated no regional lymph node metastasis, N1 indicated 1 to 3 axillary lymph node metastasis, N2 indicated 4 to 9 cases of axillary lymph node metastasis. ns: not statistically significant, *p < 0.05, and ***p < 0.001.

Subsequently, we used the UCLCAN database to investigated the GSTM2 mRNA expression in different patient groups (**Supplementary Table S1**). In **Figure 2A**, the GSTM2 expression was significantly decreased in colon cancer tissues of patients at stages 1, 2, 3, and 4 versus normal controls, while there was no significant difference between different stages. In terms of race, the GSTM2 expression was reduced in tumor tissues of Caucasian, African-american, and Asian patients versus normal controls, but there were no significant differences between distinct races (**Figure 2B**). Regarding to gender, there was no significant difference between male and female patients (**Figure 2C**). As for age, the GSTM2 expression was decreased in each age group versus normal group, and the GSTM2 expression was significantly higher in patients aged 21-40 years than 41-60 years, 61-80 years, 81-100 years (**Figure 2D**). In terms of weight, the GSTM2 expression was down-regulated in tumorous tissues from patients of different weights compared to normal tissues, while it was higher in the obese group than in the normal weight group (**Figure 2E**). As for histological subtypes, the GSTM2 expression was decreased in tumorous tissues of patients with different subtypes of colon cancer versus normal tissues, but there was no statistical difference between each two histological subtypes (**Figure 2F**). As for lymph node metastases, the GSTM2 expression in cancerous tissues from patients at N0, N1, and N2 stages was lower than in normal tissues. Still, there was no statistical difference in expression between the three groups (**Figure 2G**). Regarding to TP53 mutations, the GSTM2 was decreasingly expressed in tumor tissues from patients with wild type TP53 and mutated TP53 versus normal tissues, but the TP53 mutations did not affect the GSTM2 expression (**Figure 2H**).

At the same time, we also compared the GSTM2 protein expression in different patient subgroups based on data sets from the UALCAN database. Shown in **Supplementary Figure S1A**, the GSTM2 protein expression in tumor tissues from patients at different tumor stages was lower than normal tissue. Still, there was no significant difference in GSTM2 protein expression between each stage. In terms of races, the expression of GSTM2 protein in cancerous tissues of Caucasian, African-american, and Asian patients was lower than in normal group. The statistical results showed that GSTM2 protein expression in Asian was higher than in Caucasian (**Supplementary Figure S1B**). As for gender, the GSTM2 protein expression in tumor tissues from both male and female colon cancer patients was lower than in normal tissues, while there was no statistical difference in GSTM2 protein expression between male and female patients (**Supplementary Figure S1C**). In terms of age, the GSTM2 protein expression was decreasingly expressed in cancerous tissues from patients of different ages than in control group, but there was no statistical different between each subgroup (**Supplementary Figure S1D**). Regarding to weight, there were no differences in protein expression between distinct groups (**Supplementary Figure S1E**). As for sub-classification, the GSTM2 protein was decreasingly expressed in mucinous and non-mucinous colon cancer tissues versus normal tissues, but there was no statistical difference between each two sub-classifications (**Supplementary Figure S1F**).

### The lower GSTM2 expression predicts worse prognosis of colon cancer patients

**Figure 3:**
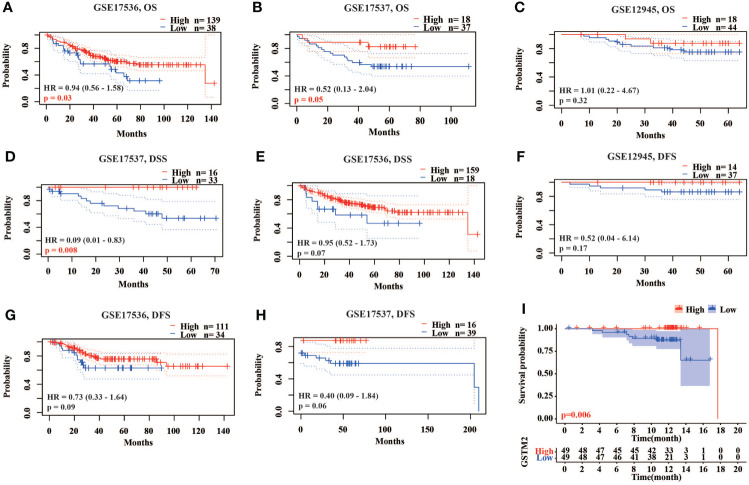
The Lower GSTM2 Expression Predicts Worse Prognosis of Colon Cancer Patients. **(A–C)** The correlation between the GSTM2 expression and OS of colon cancer patients using the GSE17536, GSE17537, GSE12945 datasets. **(D–F)** The correlation between the GSTM2 mRNA expression and DSS of colon cancer patients using the GSE17537, GSE17536, GSE12945 datasets. **(G, H)** The correlation between the GSTM2 mRNA expression and DFS of colon cancer patients using the GSE17536, GSE17537 datasets. **(I)** The correlation between the GSTM2 protein expression and OS of colon cancer patients using the dataset from CPTAC.

To clarify the functional role and prognostic value of GSTM2 in colon cancer, we further analyzed the relationship between the GSTM2 expression and prognosis of colon cancer patients using four data sets from Gene Expression Omnibus (GEO) collected by the Prognoscan database. Based on the results of three data sets named GSE17536, GSE17537, GSE12945, we demonstrated that colon cancer patients with lower expression of GSTM2 had worse overall survival rates (OS), disease-free survival (DFS) and disease specific survival rates (DSS) (**Figures 3A–H**). Also, the survival analysis revealed colon cancer patients with low GSTM2 protein expression had a shorter overall survival (**Figure 3I**). Therefore, the GSTM2 mRNA expression was potentially associated with the survival of colon cancer patients, and GSTM2 might be served as a prognostic marker for colon cancer. To explore the prognosis of GSTM2 mutant group and non-mutant group, we used the data sets from cBioportal database for analysis (n_mutant_ = 31, n_non-mutant_ = 3376). However, the results demonstrated that there was no statistical difference in survival rate between GSTM2 mutant and non-mutant group (p = 0.127) (**Supplementary Figure S2A**). We hypothesized that the negative result was due to a lack of samples in the mutant group, or that the GSTM2 mutants were not functional, thus having no effect on patient survival rates.

### Two potential reasons for the down-regulation of GSTM2 in human colon cancer

**Figure 4:**
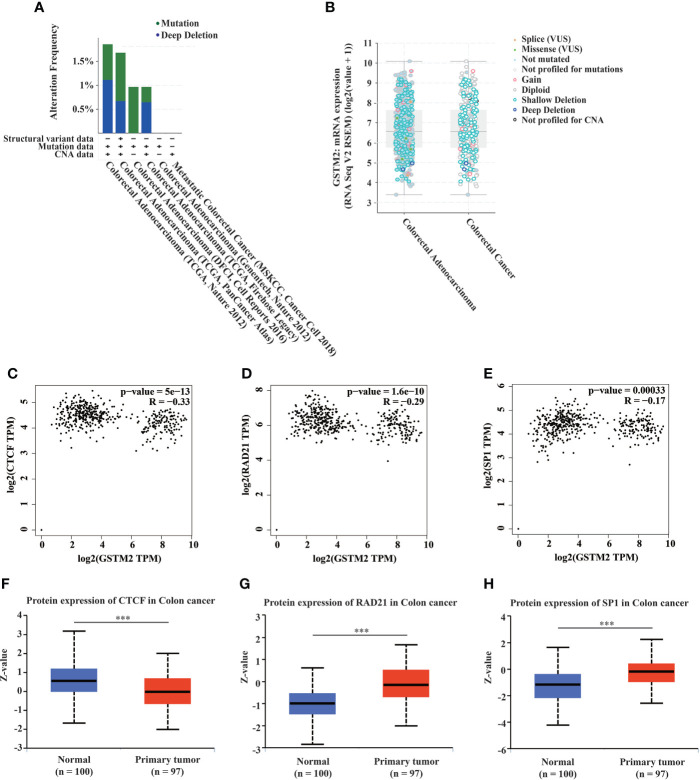
Two Potential Reasons for the Down-regulation of GSTM2 in Human Colon Cancer. **(A)** The mutation type and frequency of GSTM2 in colorectal cancer. **(B)** The GSTM2 expression in tumor tissues from colorectal cancer patients with different GSTM2 mutations. **(C–E)** The protein expression correlation between GSTM2 and CTCF, RAD21, SP1 in colon cancer. **(F–H)** The protein expression of CTCF, RAD21, SP1 in colon cancer tissues versus normal tissues. ***p < 0.001.

To explore the possible reasons for the down-regulation of GSTM2 in colon cancer, we first studied the DNA mutations of GSTM2. By analyzing the data sets from the cBioportal database (n = 4488), we found GSTM2 had a moderate mutation frequency in colon cancer patients (1%-4%), and about 50% of these mutations were deep deletion (**Figure 4A**). And, the GSTM2 mRNA expression in cancerous tissues from patients with deep deletion was lower than the median value of all types of mutations (**Figure 4B**). This result indicated that deep deletion on DNA might be one of the reasons for the down-regulation of GSTM2 expression in colon cancer tissues.

Additionally, we investigated the expression changes of the potential transcription factors of GSTM2 in colon cancer tissues versus normal tissues. We first downloaded 190 experimentally confirmed transcription factors of GSTM2 from the hTFtarget database (**Supplementary Table S2**), of which CTCF, RAD21, SP1 were identified transcription factors in colon tissues. As we knew that the transcription factors always had a correlated expression with their downstream genes, thus we used the GEPIA database to explore the expression correlation between GSTM2 and CTCF, RAD21, SP1 in colon cancer. The results showed that there were significantly negative expression correlations between the GSTM2 expression with the three transcription factors (**Figures 4C–E**), which suggested CTCF, RAD21, SP1 as potential transcription factors of GSTM2 in colon cancer. Next, we used the UALCAN database to analyze the expression of these three transcription factors in colon cancer. Shown in **Figure 4F**, the CTCF protein was expressed lower in tumor tissues than normal adjacent tissues, while RAD21, SP1 protein were expressed more in cancerous tissues than in normal tissues (**Figures 4G, H**). Therefore, of the three genes, the expression of RAD21 and SP1 in colon cancer matched the results of correlation analysis, so that RAD21 and SP1 might be the potential upstream transcription factors of GSTM2 in colon cancer. Consequently, the up-regulation of RAD21 and SP1 might be the second probable reason for the down-regulated expression of GSTM2 in colon cancer.

### The functions of GSTM2 in colon cancer might be associated with immune modulations

**Figure 5:**
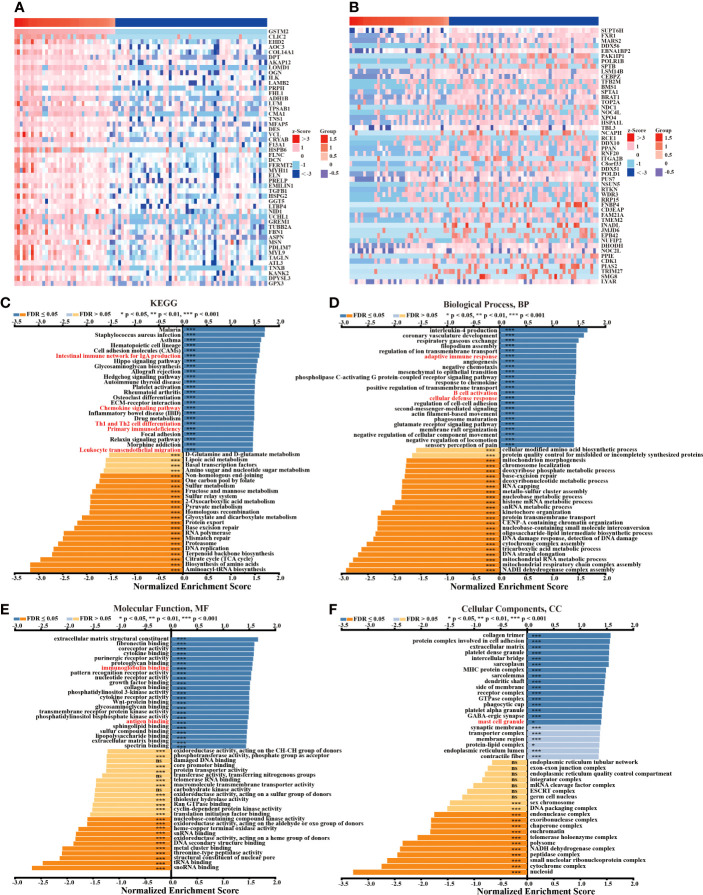
The Functions of GSTM2 in Colon Cancer might be Associated with Immune Modulations. **(A)** Heat maps of the top 50 genes positively correlated with GSTM2 expression. **(B)** Heat maps of the top 50 genes negatively correlated with GSTM2 expression. **(C–F)** GSEA analysis showing the positively and negatively correlated pathways of GSTM2. ns: not statistically significant, *p < 0.05, and ***p < 0.001.

To understand the underlying mechanism by which GSTM2 affected the development of colon cancer, we first collected a total of 5538 co-expressing mRNAs of GSTM2 from the linkedomics database (**Supplementary Table S3**). The first 50 genes positively and negatively correlated with GSTM2 were shown in **Figures 5A, B**. Then, we performed GSEA on GSTM2 to search the pathways GSTM2’s probably participating in, and the enrichment results showed that GSTM2 were most associated with immune related biological processes and pathways, such as chemokine signaling, leukocyte transendothelial migration, IgA production, Th1 and Th2 cell differentiation, primary immunodeficiency, adaptive immune response, B cell activation, defense response, immunoglobulin binding, and antigen binding (**Figures 5C–F**). These results suggested that GSTM2 might affect the tumorigenesis of colon cancer *via* regulating the immune microenvironment. Furthermore, we performed Gene Ontology (GO) and Kyoto Encyclopedia of Genes and Genomes (KEGG) enrichment analyses of GSTM2’s co-expressed genes which were downloaded from Linkedomics database (|correlation coefficient| > 0.3, false discovery rate (FDR) < 0.05). As shown in **Supplementary Figures S2B–E**, GSTM2 co-expressed genes were not only involved in tumor-related pathways, but also in immune-related pathways, such as chemokine signaling pathway and cytokine binding.

To learn more about GSTM2’s potential function in carcinogenesis, we analyzed the correlation between GSTM2 expression and the activities of key pathways (or gene signatures) involved in tumorigenesis. The results showed that GSTM2 expression was associated with the activities of signatures including cellular response to hypoxia, tumor proliferation signature, apoptosis, DNA repair, G2M checkpoint, MYC targets, TGFB, DNA replication, and degradation of ECM (**Supplementary Figure S3**).

### The GSTM2 expression was potentially related to immune infiltration in colon cancer

**Figure 6:**
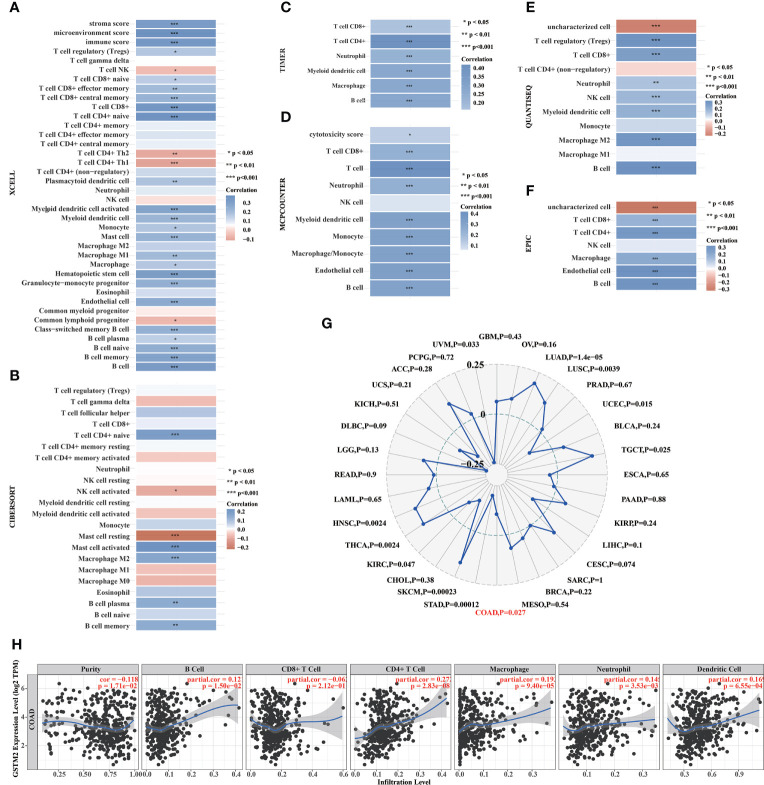
The GSTM2 Expression in Colon Cancer was Potentially Related to Immune Infiltration. **(A–F)** The correlation between the GSTM2 expression and infiltration ratios of immune cells in colon cancer as analyzed separately using the XCELL, CIBERSORT, TIMER, MCPCOUNTER, QUANTISEQ, and EPIC algorithms. **(G)** The correlation between GSTM2 expression and MSI in various tumors. **(H)** The correlations between GSTM2 expression and the infiltration levels of six classic types of immune cells in colon cancer. *p < 0.05, **p < 0.01, ***p < 0.001.

To confirm the correlation between the GSTM2 expression and immune microenvironment, we used six distinct algorithms to assess the correlation between the GSTM2 expression and immune cell infiltration in human colon cancer. As shown in **Figure 6A**, there was a significant correlation between the GSTM2 mRNA expression and stromal score, microenvironment score, and immunity score in colon cancer (p < 0.001). Meanwhile, the XCELL results revealed that the GSTM2 expression was positively correlated with the ratios of 21 types of immune cells, such as regulatory T cells (Tregs), T cell CD8+, monocyte, macrophage, endothelial cell, B cell, and was negatively correlated with the ratios of four types of immune cells, including T cell natural killer cell (NK), T cell CD4+ T helper 2 cell (Th2), T cell CD4+ T helper 1 cell (Th1), and common lymphoid progenitor. Besides, the CIBERSORT results demonstrated that the GSTM2 expression was positively correlated with the levels of T cell CD4+ naïve, mast cell activated, macrophage M2, B cell plasma, B cell memory infiltration, and negatively correlated with mast cell resting (**Figure 6B**). The results of the TIMER algorithm showed that the GSTM2 expression was highly positively correlated with the infiltration degrees of T cell CD8+, T cell CD4+, neutrophil, myeloid dendritic cell, macrophage, B cell, and other cells (**Figure 6C**). The MCPCOUTER results uncovered that the GSTM2 expression was significantly positively correlated with the infiltrating ratios of T cell CD8+, T cell, neutrophil, myeloid dendritic cell, monocyte, macrophage, endothelial cell, and B cell (**Figure 6D**). The QUANTISEQ results showed that the GSTM2 expression was positively correlated with the infiltration levels of Tregs, T cell CD8+, neutrophil, NK cell, myeloid dendritic cell, macrophage M2, and B cell (**Figure 6E**). Similarly, the EPIC algorithm results showed that the GSTM2 expression was positively correlated with the infiltration degrees of T cell CD8+, T cell CD4+, macrophage, endothelial cell, and B cell (**Figure 6F**). Taken together, these results disclosed that the GSTM2 mRNA expression was correlated to the immune scores, and universally associated with the infiltration levels of five types of immune cells, including B cell, T cell CD8+, T cell CD4+, macrophage, dendritic cell, which further indicated that GSTM2 was a potentially regulatory gene for immune microenvironment of colon cancer. In colon cancer, MSI was highly associated with immune cell infiltration and patients’ clinical response to immunotherapy (21, 22), thus we evaluated the correlation between GSTM2 expression and MSI in various cancers. As a result, GSTM2 expression was negatively associated with MSI in colon cancer (**Figure 6G**). This result supports the conclusion of our study that GSTM2 may be involved in the regulation of immune microenvironment in colon cancer, and also indicates that GSTM2 expression may have an impact on immunotherapy. After that, we used TIMER database to analyze the relationship between GSTM2 expression and the infiltration levels of six kinds of classical immune cells in TCGA colon cancer patients. As shown in **Figure 6H**, GSTM2 expression was significantly positively correlated with the infiltration degrees of B cells, CD4+T cells, macrophages, neutrophils and dendritic cells, which was consistent with **Figures 6A–F** results calculated by the six algorithms.

### The immunohistochemical results confirmed that GSTM2 was decreasingly expressed in tumor tissues versus normal adjacent tissues based on our own self-built Chinese cohort

**Figure 7:**
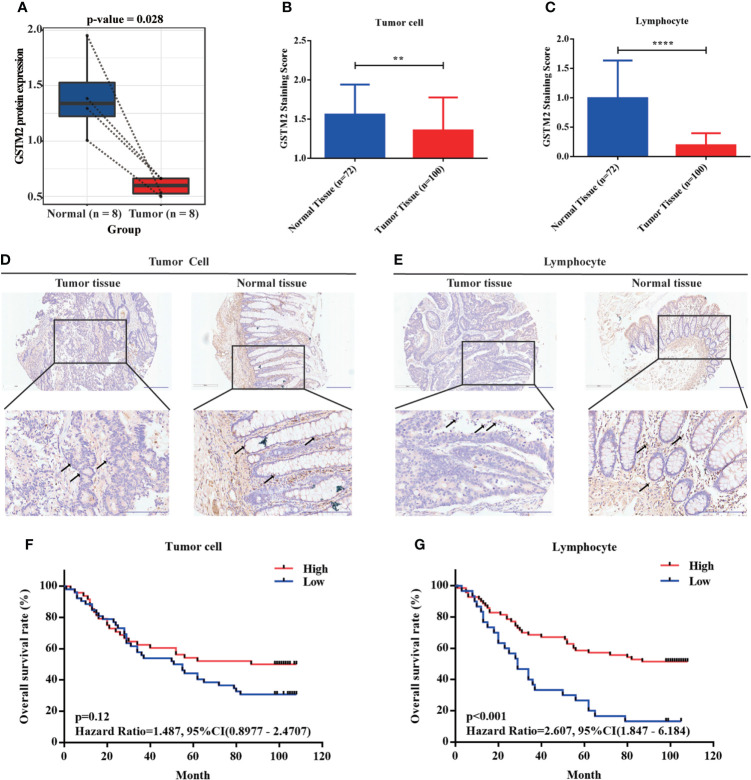
The Immunohistochemical Results Disclosed that GSTM2 was Decreasingly Expressed in Tumor Tissues versus Normal tissues Based on our Self-built Chinese Cohort. **(A)** The GSTM2 expression was evaluated in 4 paired colon cancer tissues and adjacent normal tissues using our own proteomic data. **(B)** The GSTM2 staining score in colon cancer versus normal tissue. **(C)** The GSTM2 staining score in lymphocyte within colon cancer tissue versus normal tissue. **(D, E)** Representative images of immunohistochemical staining of tumor cell and lymphocyte within tumor tissues and normal tissues. The arrows identify representative lymphocytes or tumor cells. **(F, G)** The correlation between the GSTM2 protein expression with the OS of colon cancer patients based on our self-built Chinese cohort. **p < 0.01 and **** p < 0.0001.

To confirm the GSTM2 expression in colon cancer, we first analyzed the GSTM2 protein expression in human colon cancer tissues and normal adjacent tissues using our own proteomic data (n = 8, samples of the same stage were pooled into one sample) (23). As a result, the expression of GSTM2 protein was significantly lower in cancerous tissues compared to their paired non-tumor tissues (**Figure 7A**). Next, we further detected the GSTM2 protein expression in our self-built Chinese cohort (n_tumor_ = 100, n_normal_ = 72) using the immunohistochemical assay (**Supplementary Data**), and the patient clinical information were detailed in **Table 1**. When solely calculating the colonic cells, we observed the staining score of GSTM2 (stain intensity timed the percentage of stained cells) in cancerous cells was significantly lower than normal adjacent cells (p = 0.0015) (**Figure 7B**). Since GSTM2 is potentially related to the regulation of immune microenvironment in colon cancer, and the previous studies have also shown that the expression changes of functional genes in immune cells may affect the tumorigenesis, we then analyzed the expression changes of GSTM2 in the infiltrating lymphocytes within tumor tissues. As a result, the staining intensity of GSTM2 in the lymphocytes within the cancerous tissues was significantly lower than the lymphocytes within the normal tissues (p = 0.0001) (**Figure 7C**). The **Figures 7D, E** were the representative pictures of immunohistochemical detection of GSTM2 in cancer cells or lymphocytes within the tumor tissues and normal adjacent tissues. Furthermore, we also detected GSTM2 level in different grades, T stages, N stages and M stages of colon cancer, and found that the GSTM2 level in tumor cells and lymphocytes decreased with the increase of M stages (**Supplementary Figure S4**), suggesting that GSTM2 might be associated with metastasis.

**Table 1:** The characteristics of 104 colorectal cancer patients in our study.

	Type	Patients
**Age**	≦63	32 (30.7%)
	>63	66 (63.5%)
	–	6 (5.8%)
**Sex**	Male	58 (55.8%)
	Female	45 (43.3%)
	–	1 (0.9%)
**Pathological type**	Adenocarcinoma	53 (51.0%)
	Tubular adenocarcinoma	44 (42.3%)
	Mucinous adenocarcinoma	7 (6.7%)
**Pathological grade**	I- II	6 (5.8%)
	I- III	1 (0.9%)
	II- III	33 (31.8%)
	II	51 (49.0%)
	III	13 (12.5%)
**T-stage**	T1	1 (0.9%)
	T2	4 (3.9%)
	T3	81 (77.9%)
	T4a	7 (6.7%)
	T4b	7 (6.7%)
	–	4 (3.9%)
**N-stage**	N0	64 (61.6%)
	N1a	12 (11.5%)
	N1b	14 (13.5%)
	N2a	10 (9.6%)
	N2b	2 (1.9%)
	–	2 (1.9%)
**M-stage**	M0	101 (97.1%)
	M1b	3 (2.9%)
**Survival time (month)**	≦32	39 (37.5%)
	>32	65 (62.5%)

Also, we analyzed GSTM2 mRNA expression in individual cells using the data set of Li’s work published in Nature Genetics (24). The results revealed that compared with certain cells from normal tissues, GSTM2 expression was statistically reduced in stem cells (p = 0.008), and had a decreasing trend in fibroblasts (p = 0.311) and B cells (p = 0.13) from colorectal cancer tissues (**Supplementary Table S4**). As is well-known, stem cells are a kind of primitive cells with the self-renewal ability and multidirectional differentiation potential, which is the origin of cell proliferation. Tumor stem cells have the properties of initiating tumors, promoting metastasis, enhancing drug resistance (25). The lower expression of GSTM2 in stem cells further illustrates this gene’s potential importance and functions in tumorigenesis of colon cancer.

At the same time, we also evaluated the effects of GSTM2 protein expression on patient survival after grouping the patients into two groups based on the best cutoff value of GSTM2 expression (the best cutoff value of GSTM2 expression in tumor cell group was 1, and in lymphocyte group was 0.075). When we analyzed the GSTM2 expression in tumor cells, we found that the patients with lower GSTM2 immunohistochemical scores frequently had poorer OS (p = 0.12, HR = 1.487, 95%CI (0.9877 - 2.4707) (**Figure 7F**). Similarly, when we analyzed the GSTM2 expression in infiltrating lymphocytes, the patients with lower GSTM2 immunohistochemical scores also had worse OS (p < 0.001, HR = 2.607, 95%CI (1.847 - 6.184) (**Figure 7G**). We also performed univariate and multivariate Cox regression analyses of GSTM2 protein expression and clinical characteristics, and the results showed that the p value of GSTM2 protein level in infiltrating lymphocytes within tumor tissues was less than 0.05, which indicated that GSTM2 was potentially an independent prognosis factor for colon cancer (**Table 2**).

**Table 2:** Univariate and multivariate analyses identified GSTM2 level in lymphocytes as an independent prognostic factor for colon cancer.

	Univariate analysis			Multivariate analysis		
	p-value	Hazard Ratio	95% confidence interval	p-value	Hazard Ratio	95% confidence interval
**GSTM2 level in tumor cells**	0.131	0.671	0.400-1.126	0.402	0.775	0.428-1.405
**GSTM2 level in lymphocytes**	<0.001	0.373	0.222-0.628	0.005	0.424	0.234-0.767
**Gender**	0.42	1.24	0.735-2.094	0.023	2.078	1.108-3.897
**Age**	0.039	2.048	1.037-4.044	0.022	2.505	1.140-5.504
**Pathological grade**	0.107	1.748	0.886-3.446	0.034	2.307	1.064-5.003
**T**	<0.001	2.171	1.439-3.276	<0.001	2.328	1.521-3.563
**N**	<0.001	1.583	1.277-1.962	<0.001	1.831	1.382-2.426
**M**	<0.001	15.024	4.170-54.127	0.028	6.887	1.231-38.544

Additionally, we hoped to find out which medicines GSTM2 expression was linked to their resistance. We downloaded drug-resistance data sets from Genomics of Drug Sensitivity in Cancer (GDSC) database, exploring drug resistance relevant to GSTM2 expression, but no statistical result was found (correlation coefficient >0.2 or <-0.2, p<0.05) (**Supplementary Table S5**).

To sum up, the above results confirmed the findings we discovered based on the public data, demonstrating that GSTM2 were lower expressed in colon cancer tissues versus normal tissues and its expression were potentially relevant to the patient prognosis (OS).

### The mechanism underlying GSTM2 in colon cancer might be associated with the functions of PCDH17 mutations

**Figure 8:**
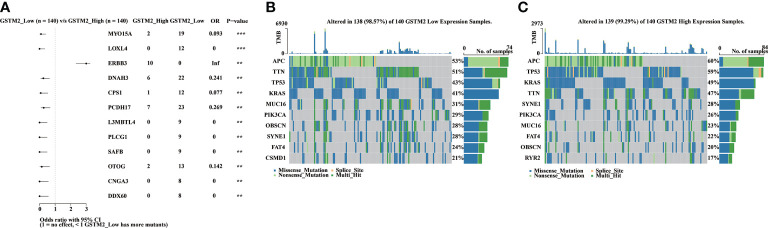
The Mechanism Underlying GSTM2 in Colon Cancer might be Associated with the Functions of PCDH17 mutations. **(A)** The forest map showing differential mutant genes between GSTM2 high and low expression groups. **(B, C)** The waterfall maps showing genes’ mutation frequencies in each group. **p < 0.01 and ***p < 0.001.

In addition, we analyzed the differences in gene mutations between GSTM2-high-expression and GSTM2-low-expression groups using the data sets from TCGA database. To further clarify the mechanism of GSTM2 in tumorigenesis, we analyzed genome-wide mutation differences in GSTM2-high-expression and low-expression colon cancer patients. We found that the mutation frequency of ERBB3 were drastically increased in GSTM2 high-expressed patients, while the mutations of MYO15A, LOXL4, DNAH3, CPS1, PCDH17, L3MBTL4, PLCG1, SAFB, OTOG, CNGA3, and DDX60 were significantly elevated in GSTM2-low-expression patients (**Figures 8A–C**). Among these mutations, PCDH17 has been identified as a driver gene that induces colon cancer tumorigenesis (26). Therefore, we speculated that the functions of GSTM2 in colon cancer carcinogenesis might be related to PCDH17 mutation.

## Discussion

The GSTM family encodes a series of detoxifying enzymes that promote the metabolism of toxic substances, thereby may play an important role in tumor suppression. Some reports have revealed that the GSTM enzymes also participate in the metabolism of tumor chemotherapeutic drugs, and protection of organelle or cells from stress injures (27). GSTM2 encodes a protein with GST structure domain that plays important roles in various biological activities (7). In addition to detoxification, GSTM2 is also a transporter that can carry lipophilic molecules such as cholic acid, bilirubin, hormones, thyroid hormones, steroids, and a series of exogenous compounds and engage in the production of hormones, such as prostaglandins (28). GSTM2 has been discovered to participate in ASK1, JNK, and P38-MAPK pathways in organisms (29). Several studies have proclaimed that the GSTM1 expression is closely related to the poorer prognosis of colorectal cancer, breast cancer, and lung cancer (30–32). However, the available researches on GSTM2 are relatively insufficient.

Guo E, et al. (13)have demonstrated that the low expression of GSTM2 in colon cancer is related to the “better” prognosis of patients using a bioinformatics analysis. Wonderingly, the lower expression of functional genes is usually associated with “poorer” prognosis in general, thus we speculated that some factors, such as sample size, race, tumor subtype, and age, might have influenced the previous result. Using three independent data sets and a data set of self-constructed Chinese cohort, our study revealed that the lower expression of GSTM2 was associated with the poorer patient outcomes. Compared with the previous study, our study has the following innovative, expanded and in-depth discoveries: First, we not only revealed the expression of GSTM2 and its relationship with prognosis through public data, but also verified the results through our self-built Chinese cohort (n _tumor_ = 104, n _normal_ = 74). We confirmed the GSTM2 expression in Chinese patients and its effect on the occurrence of colon cancer. Second, we uncovered the possibility that GSTM2 might regulate tumorigenesis *via* modulating the immune microenvironment. To our best knowledge, this is the first time that the potential role of GSTM2 in tumor immunity has been disclosed. Third, we identified two potential causes of down-regulation of GSTM2 expression in colon cancer.

The tumor microenvironment of colon cancer plays an extremely important role in tumorigenesis. Our results revealed that the GSTM2 expression was probably positively associated with the infiltration ratios of most immune cells in colon cancer, such as CD8+ T cells. It has been reported that CD8+ T cells can kill tumor cells, affecting the prognosis of colon cancer and immunotherapy responses (33, 34). Therefore, the patients with low GSTM2 expression might have low infiltrating ratios of T cell CD8+, thereby the tumor initiation may be promoted.

We predicted the potential upstream transcription factors of GSTM2 in colon cancer tissues. First, we discovered three GSTM2 transcription factors in colon tissues using the TFtarget database, and we suspected that in colon cancer tissues, these transcription factors might probably launch GSTM2’s transcription. Because transcription factors are frequently connected with the expression of target genes, we used transcriptome data of colon cancer tissues to determine which transcription factors were more likely to co-express with GSTM2. Eventually, we identified RAD21 and SP1 as potential transcription factors of GSTM2 in colon cancer. RAD21 encodes a subunit of cohesin, and mainly involved in modifying the adhesion and separation of sister chromatids, thus regulating gene transcription and DNA damage repairing. Numerous studies have revealed that RAD21 is over expressed in a variety of tumors, such as colorectal cancer, non small cell lung cancer, and breast cancer (35–37). Besides, It has also been proven that the higher RAD21 expression in tumor tissues is associated with shorter DSS of patients with colorectal cancer. Also, the higher expression of RAD21 in patients promotes the resistance of chemoradiotherapy and chemotherapy (36). All in all, our results are consistent with the previous studies, and the function of RAD21 in colon cancer further indicates that GSTM2 may be involved in tumor formation as a suppressor.

## Conclusion

To sum up, our findings revealed that GSTM2 was decreasingly expressed in colon cancer tissues versus normal adjacent tissues, and its lower expression was associated with poorer prognosis of patients. Furthermore, we uncovered the potential immunological functions of GSTM2 in colon cancer. Our findings here suggest that GSTM2 may serve as a prognostic biomarker for colon cancer, and as a potential drug target for immunotherapy.

## Data availability statement

The original contributions presented in the study are included in the article/**Supplementary Material**. Further inquiries can be directed to the corresponding authors.

## Ethics statement

The studies involving human participants were reviewed and approved by Ethics Committee of Taizhou Hospital. The patients/participants provided their written informed consent to participate in this study.

## Author contributions

Study concept and design: WZ, DT. Acquisition of data: WZ, LinL, YZ. Analysis and interpretation of data: YS, LieL. Statistical analysis: WZ, YC, SN, WC. Drafting of the manuscript: WZ, YS. Critical revision and final approval of the manuscript: all authors. Obtained funding: MT, YD, WZ. Study supervision: XG, MT. All authors contributed to the article and approved the submitted version.

## Funding

This study was supported by the National Natural Science Foundation of China (No. 82003172), the Postdoctoral Science Foundation of China (No. 2020M673065), the Guangdong Basic and Applied Basic Research Foundation (No. 2019A1515111138, No.2021A1515111071).

## Acknowledgments

The authors would like to thank Taizhou Hospital for providing patients’ paraffin blocks, and Shenzhen People’s Hospital for providing fund support for the determination of experimental data.

## Conflict of interest

The authors declare that the research was conducted in the absence of any commercial or financial relationships that could be construed as a potential conflict of interest.

## Publisher’s note

All claims expressed in this article are solely those of the authors and do not necessarily represent those of their affiliated organizations, or those of the publisher, the editors and the reviewers. Any product that may be evaluated in this article, or claim that may be made by its manufacturer, is not guaranteed or endorsed by the publisher.
