# Supplementary material for: Integrated computer analysis and a self-built Chinese cohort study identified GSTM2 as one survival-relevant gene in human colon cancer potentially regulating immune microenvironment
Source: Front Oncol. 2022 Oct 3;12:881906. doi: 10.3389/fonc.2022.881906 (PMC9574330; doi:10.3389/fonc.2022.881906)
Supplement: Supplementary file 1 [file DataSheet_1.pdf]

Supplementary Figure S3

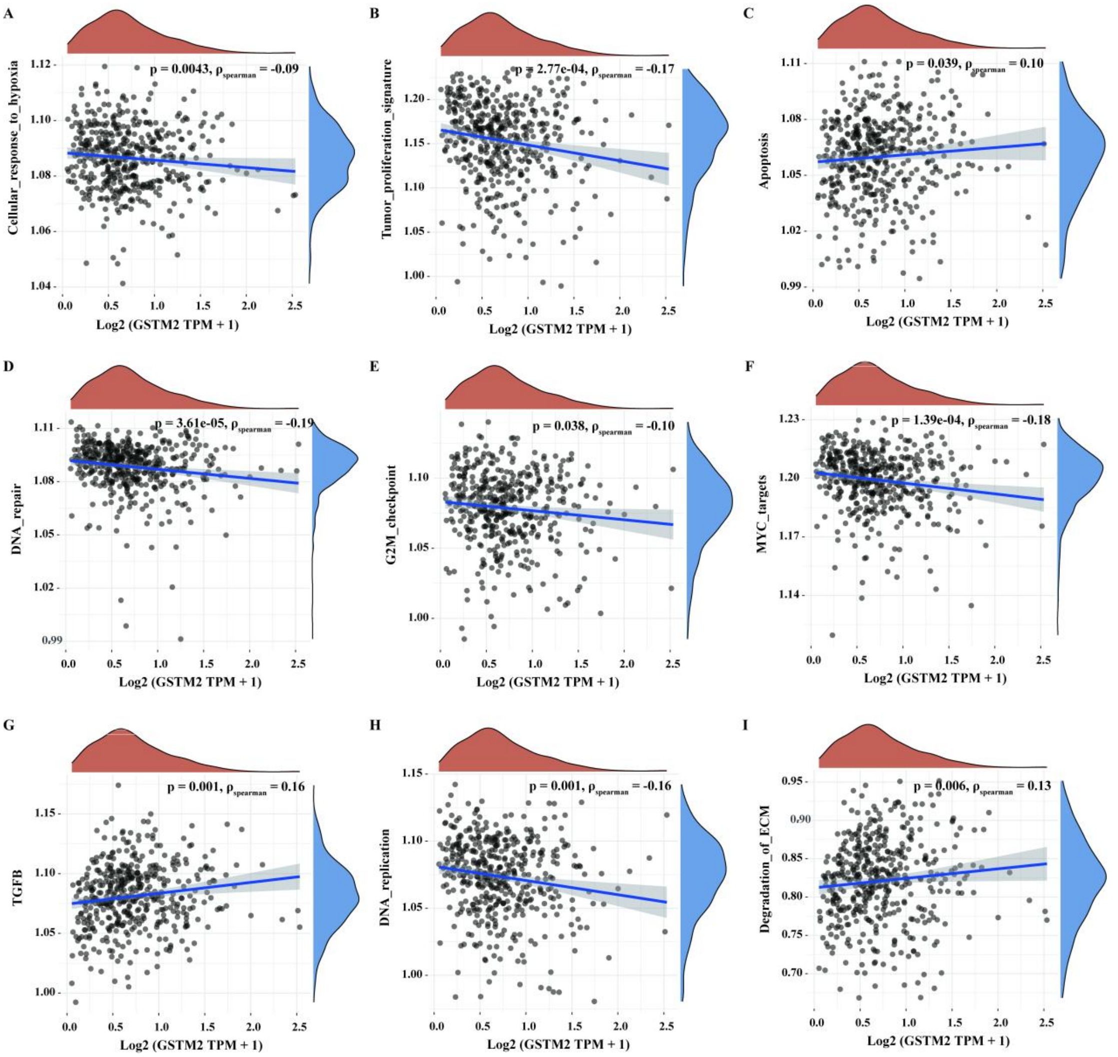

Supplementary Figure S3 GSTM2 expression was significantly correlated with the activities of key pathways involved in tumorigenesis in colon cancer. GSTM2 expression was associated with the activities of signatures in colon cancer, including (A) cellular response to hypoxia, (B) tumor proliferation signature, (C) apoptosis, (D) DNA repair, (E) G2M checkpoint, (F) MYC targets, (G) TGFB, (H) DNA replication, and (I) degradation of ECM.
