# Supplementary material for: Integrated computer analysis and a self-built Chinese cohort study identified GSTM2 as one survival-relevant gene in human colon cancer potentially regulating immune microenvironment
Source: Front Oncol. 2022 Oct 3;12:881906. doi: 10.3389/fonc.2022.881906 (PMC9574330; doi:10.3389/fonc.2022.881906)
Supplement: Supplementary file 2 [file DataSheet_2.pdf]

Supplementary Figure S4

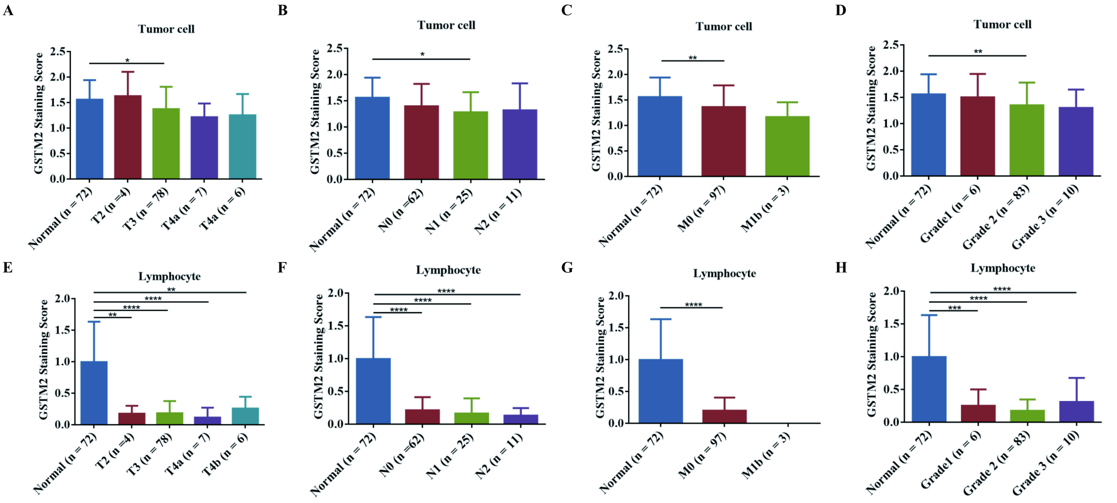

Supplementary Figure S4 GSTM2 expression in different grades, T stages, N stages and M stages of colon cancer. GSTM2 expression in (A-D) tumor cells, and (E-H) lymphocytes of different grades, T stages, N stages and M stages of colon cancer.
