# Supplementary figures and images for: Integrated computer analysis and a self-built Chinese cohort study identified GSTM2 as one survival-relevant gene in human colon cancer potentially regulating immune microenvironment
Source: Front Oncol. 2022 Oct 3;12:881906. doi: 10.3389/fonc.2022.881906 (PMC9574330; doi:10.3389/fonc.2022.881906)

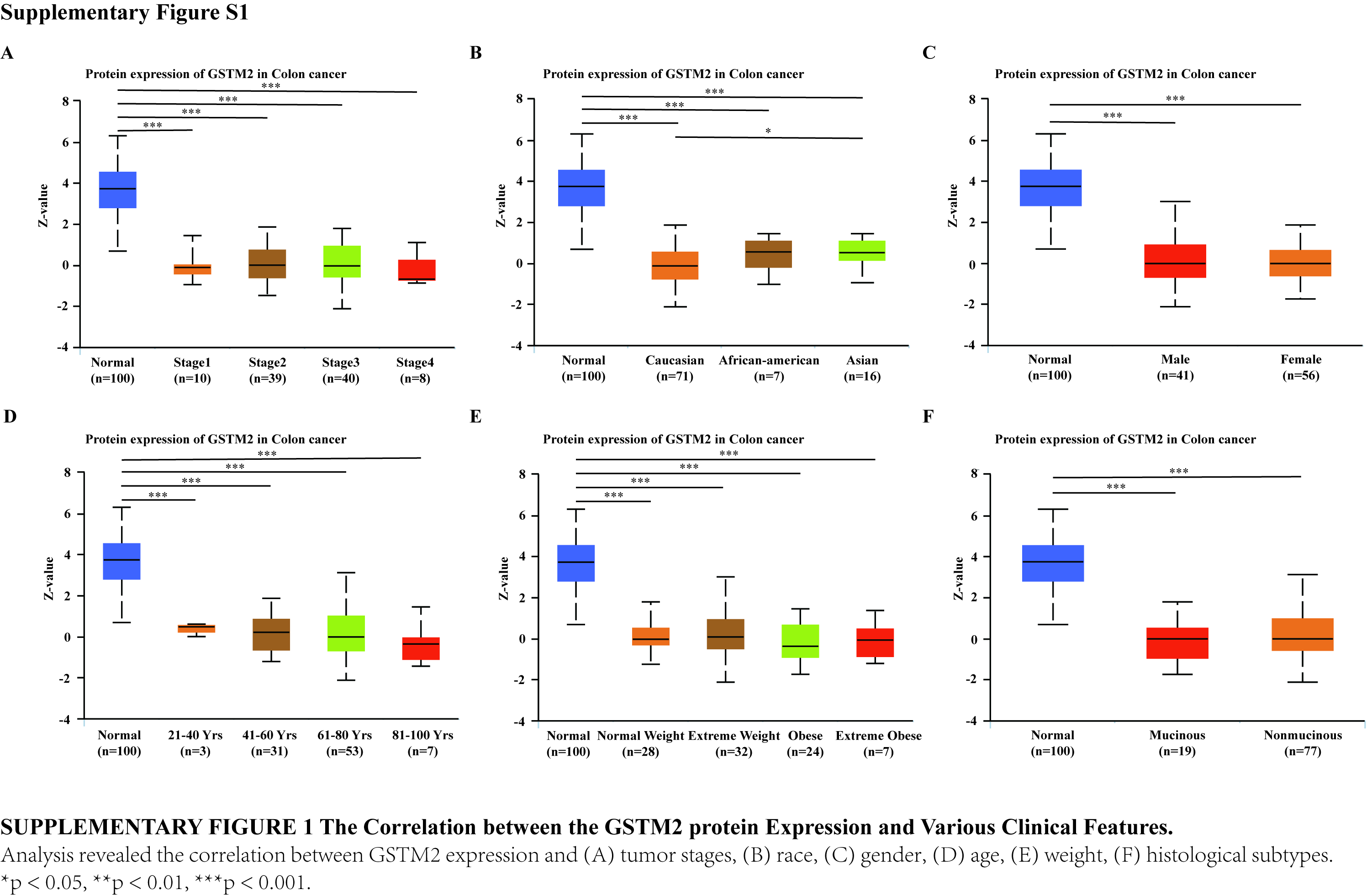

Supplement: Supplementary file 3 [file Image_1.tif]

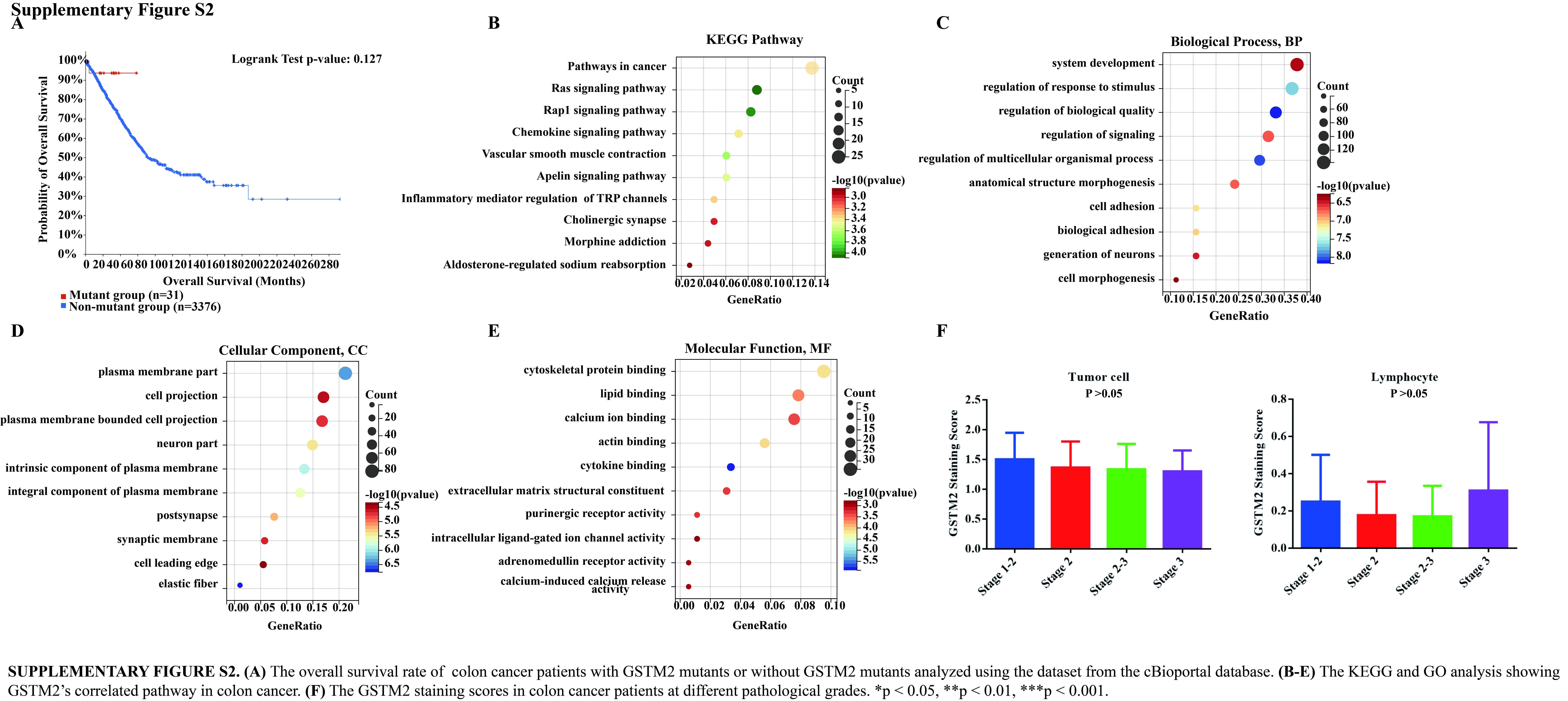

Supplement: Supplementary file 4 [file Image_2.jpeg]
